# Ventricular arrhythmia burden in ICD patients during the second wave of the COVID-19 pandemic

**DOI:** 10.1007/s00392-023-02320-2

**Published:** 2023-10-30

**Authors:** Benjamin Rath, Florian Doldi, Kevin Willy, Christian Ellermann, Julia Köbe, Fatih Güner, Florian Reinke, Philipp Sebastian Lange, Gerrit Frommeyer, Lars Eckardt

**Affiliations:** Department of Cardiology II-Electrophysiology, University Hospital Münster, Albert-Schweitzer-Campus 1, 48149 Münster, Germany

**Keywords:** COVID-19, ICD, Ventricular arrhythmia, VT, VF

## Abstract

**Aim:**

COVID-19 has been associated with cardiovascular complications including ventricular arrhythmias (VA) and an increased number of out-of-hospital cardiac arrests. Nevertheless, several authors described a decrease of VA burden in patients with an implantable defibrillator (ICD) during the first wave of the COVID-19 pandemic. The objective of this study was to determine if these observations could be transferred to later periods of the pandemic as well.

**Methods:**

We retrospectively analyzed a total of 1674 patients with an ICD presenting in our outpatient clinic during the second wave of the COVID-19 pandemic and during a control period for the occurrence of VA requiring ICD interventions.

**Results:**

Seven hundred ninety-five patients with an ICD had a device interrogation in our ambulatory clinic during the second wave of the COVID-19 pandemic compared to eight hundred seventy-nine patients in the control period. There was significant higher amount of adequate ICD therapies in the course of the COVID-19 period. Thirty-six patients (4.5%) received in total eighty-five appropriate ICD interventions during COVID-19, whereas only sixteen patients (1.8%) had sustained VA in the control period (*p* = 0.01).

**Conclusion:**

In contrast to the first wave of COVID-19, which was characterized by a decrease or least stable number of ICD therapies in several centers, we found a significant increase of VA in ICD patients during the second wave of COVID-19. Possible explanations for this observation include higher infectious rates, potential cardiac side effects of the vaccination as well as personal behavioral changes, or reduced utilization of medical services.

## Introduction

Since the first emergence of COVID-19 in late 2019, there has been a vastly documented surge in cases of infected patients followed by various vaccines and treatment attempts aimed at reducing the infectious rate and possible severe courses of the disease. COVID-19 primarily affects the respiratory system, but major cardiovascular complications including ventricular arrhythmias (VA) have been reported in hospitalized patients [1–3]. Despite or as a result of a decline in cardiovascular hospitalizations [4], several countries reported an increased number of out-of-cardiac-arrests (OHCA) during the COVID-19 pandemic [5, 6]. The risk of VA arises in settings of elevated sympathetic tone like illness, physical or emotional distress [7]. Historically, a significant increase of myocardial infarctions and VA has been observed during earthquakes or terrorists attacks [8, 9]. Patients with an implantable cardioverter defibrillator (ICD) are naturally more prone to develop VA because of their underlying structural or electrical heart disease. The influence of the COVID-19 pandemic on VA burden in ICD patients has been described by several authors in different regions so far [10–13]. Of note, despite the abovementioned considerations, a reduction or at least no increase of ICD interventions has been observed in the majority of these collectives [10–12]. The authors assumed an increased sedentary behavior due to extensive lockdown measures as the most probable cause for this observation. However, all these observations referred to the first wave of COVID-19 in late 2019 and early 2020. We, therefore, sought to determine if these results could also be observed in later periods of the pandemic.

## Methods

### Study cohort and design

We retrospectively analyzed all patients presenting to our outpatient clinic either for a regular check of their ICD or for an unplanned visit (e.g., because of an ICD shock). Patients were screened individually for the occurrence of sustained ventricular arrhythmias (VF or VT) requiring appropriate ICD interventions [antitachycardia pacing (ATP) or ICD shock]. Patients were examined in our outpatient clinic either during the second wave of the COVID-19 pandemic in Germany (15 November 2020–15 March 2021) or during a control period (15 November 2018–15 March 2019). As routine follow-up of ICD patients regulary takes place every 4 months in our clinic, the screened time period for the occurrence of VA in the majority of patients was 4 months since the preceding device interrogation. In patients with longer control intervals or unplanned visits (e.g., as a result of ICD shock), only VA within the last 4 months were recorded to ensure comparability between groups. Programming of specific ICD parameters such as intervention heart rate or detection intervals was left to the physician’s discretion. The study was approved by the local ethics committee.

### Statistical analysis

For descriptive statistics, continuous data were presented as means with standard deviation (SD) and medians with interquartile ranges (IQR), respectively. Categorical data were presented as proportions. Normality of data distribution was assessed using the Shapiro–Wilk test. Comparisons between groups were performed using the Pearson’s Chi-squared test for categorical variables, and Student’s *t* test or Mann–Whitney *U* test for unpaired continuous variables, and Wilcoxon rank-sum test for paired variables, according to data distribution. Logistic regression models with odds ratios (OR) and 95% confidence intervals (CI) were used to assess the risk of an ICD intervention. A *p* value of < 0.05 was defined as statistically significant. The statistical software used for data analysis and visualization was R version 1.4.1717 (The R Foundation for Statistical Computing, Vienna, Austria).

## Results

A total of 795 patients with an ICD had a device interrogation in our ambulatory clinic during the second wave of the COVID-19 pandemic compared to 879 patients in the control period. Baseline characteristics of all patients are summarized in Table 1. Six hundred ninety-nine (71.7%) of these patients had device interrogation both during COVID-19 and the control period.

**Table 1:** Baseline characteristics

	COVID-19 (11/20–03/21)	Control period (11/18–03/19)	*p* value
*n*	795 (47.5%)	879 (52.5%)	
Age	64.5 [41.2, 72.2]	61.8 [51.9, 78.4]	0.64
Sex (male)	568 (71.4%)	655 (74.5%)	0.82
ICD type			
Transvenous ICD	595 (74.8%)	662 (75.3%)	0.09
CRT-ICD	110 (13.8%)	142 (16.1%)	
Subcutanous ICD	90 (11.3%)	75 (8.5%)	
Cardiomyopathy			
Ischemic	436 (54.8%)	558 (63.5%)	0.98
Non ischemic	254 (31.9%)	233 (26.5%)	
Other	105 (13.2%)	88 (10.0%)	
Antiarrhythmic medication			
ß-blocker	629 (79.1%)	659 (76.1%)	0.91
Amiodarone	56 (7.0%)	71 (8.1%)	0.11
Flecainide	16 (2.0%)	19 (2.2%)	0.38
Sotalol	8 (1.0%)	12 (1.4%)	0.06

Overall, 49 patients (5.0%) received in total 119 appropriate ICD interventions due to sustained VA. Baseline characteristics of these patients are listed in Table 2. Forty (81.6%) of these patients had follow-up during both COVID-19 and the control period. The most common arrhythmia was monomorphic ventricular tachycardia (mVT) (46.2%) followed by ventricular fibrillation (VF) (31.1%) and polymorphic ventricular tachycardia (pVT) (22.7%)**.** Ischemic cardiomyopathy (ICM) was the underlying heart disease in more than half of these patients (53.8%) and 26.9% were treated with antiarrhythmic drugs (AAD), predominately amiodarone (23.1%). Despite a smaller overall number of investigated ICD recipients, there was a significant higher amount of patients requiring adequate ICD therapies during the COVID-19 period. Thirty-six patients (4.5%) received in total eighty-five appropriate ICD interventions during the second wave of COVID-19, whereas only sixteen patients (1.8%) had thirty-four episodes of sustained VA in the control period (OR 2.69, 95% CI 1.50, 5.01, *p* = 0.01). Three of these patients received appropriate ICD therapies in both time periods. Baseline characteristics were similar in both groups. Distribution of arrhythmia type (mVT, pVT, VF) and ICD treatment (shock vs. ATP) were similar in both groups as well. No correlation was found between use of AAD, respectively, underlying heart disease or type of ICD and arrhythmic risk during or before COVID-19. There was no significant difference of inappropriate ICD interventions during COVID-19 and the control period (Table 3). This applies both to lead malfunction and inappropriate detection of supraventricular tachycardia (SVT). When focusing only on the 699 overlapping patients with ICD interrogation during both periods (Table 4), we still found a significant higher number of patients with appropriate ICD therapies during COVID-19 compared to the control period (OR 0.48 CI 0.26, 0.91 *p* = 0.03, Table 5).

**Table 2:** Baseline characteristics of patients with appropriate ICD therapies

	COVID-19 (11/20–03/21)	Control period (11/18–03/19)	*p* value
*n*	36 (4.5%)	16 (1.8%)	0.01
Age	63.5 [52.8, 71.5]	63.5 [43.2, 71.2]	0.88
Sex (male)	29 (80.6%)	13 (81.2%)	1.00
ICD type			
Transvenous ICD	24 (66.7%)	14 (87.5%)	0.23
CRT-ICD	8 (22.2%)	2 (12.5%)	
Subcutanous ICD	4 (11.1%)	0 (0.0%)	
Treated VA episodes	85	34	0.25
mVT	38 (44.7%)	17 (50%)	
pVT	19 (22.4%)	8 (23.5%)	
VF	28 (32.9%)	9 (26.5%)	
Termination with ATP	26 (30.6%)	15 (44.1%)	
Termination with ICD shock	59 (69.4%)	19 (55.9%)	
Cardiomyopathy			
Ischemic	18 (50.0%)	10 (62.5%)	0.26
Non-ischemic	11 (30.6%)	3 (18.8%)	
Other	7 (19.4%)	3 (18.8%)	
Antiarrhythmic medication			
ß-blocker	35 (97.5%)	15 (93.8%)	1.00
Amiodarone	9 (25.0%)	3 (18.8%)	0.89
Flecainide	0 (0.0%)	2 (12.5%)	0.17

**Table 3:** Inappropriate ICD interventions

	COVID-19 (11/20–03/21)	Control period (11/18–03/19)	*p* value
n	5 (0.6%)	6 (0.7%)	0.14
SVT	1	2	0.98
Lead malfunction	4	4	0.82
ICD type			0.71
Transvenous ICD	3	3	
CRT-ICD	1	2	
Subcutanous ICD	1	1	
Cardiomyopathy			0.12
Ischemic	3	4	
Non-ischemic	1	1	
Other	1	1

**Table 4:** Baseline characteristics of overlapping patients

	COVID-19	Control period	*p* value
	(11/20–03/21)	(11/18–03/19)	
*n*	699	699	
Age	63.5	61.5	
Sex (male)	482 (69.0)	482 (69.0)	
ICD type			
Transvenous ICD	517 (74.0%)	520 (74.4%)	0.90
CRT-ICD	99 (14.2%)	97 (13.9%)	
Subcutanous ICD	83 (11.9%)	82 (11.7%)	
Cardiomyopathy			
Ischemic	367 (52.5%)	367 (52.5%)	
Non-ischemic	231 (33.0%)	231 (33.0%)	
Other	101 (14.4%)	101 (14.4%)	
Antiarrhythmic medication			
ß-blocker	566 (81.0%)	561 (80.3%)	0.79
Amiodarone	48 (6.9%)	51 (7.3%)	0.83
Flecainide	12 (1.7%)	14 (2.0%)	0.84
Sotalol	7 (1.0%)	9 (1.3%)	0.80

**Table 5:** Baseline characteristics of overlapping patients with appropriate ICD therapies

	COVID-19	Control period	*p* value
	(11/20–03/21)	(11/18–03/19)	
*n*	30 (4.3%)	15 (2.1%)	**0.03**
Age	62.2	60.8	0.22
Sex (male)	21 (70.0%)	12 (80%)	0.21
ICD type			
Transvenous ICD	21 (70.0%)	13 (86.6%)	0.18
CRT-ICD	7 (23.3%)	2 (13.3%)	
Subcutanous ICD	2 (6.7%)	0 (0.0%)	
Treated VA episodes	71	31	0.49
mVT	32 (45.1%)	14 (45.2%)	
pVT	12 (16.9%)	8 (25.8%)	
VF	27 (38.0%)	9 (29.0%)	
Termination with ATP	20 (28.2%)	12 (38.7%)	0.17
Termination with	51 (71.8%)	19 (61.3%)	0.12
ICD shock			
Cardiomyopathy			
Ischemic	15 (50%)	9 (60%)	0.09
Non-ischemic	9 (30.0%)	3 (20%)	
Other	6 (20.0%)	3 (20%)	
Antiarrhythmic medication			
ß-blocker	29 (96.7%)	15 (100%)	0.47
Amiodarone	7 (23.3%)	3 (20%)	
Flecainide	0 (0.0%)	2 (13.3%)	

*p* < 0.05

## Discussion

This study identified an increased burden of VA in patients with an ICD during the second wave of the COVID-19 pandemic in Germany. In the past, a correlation between dramatic events like natural disasters or terroristic attacks and cardiac arrhythmias could be observed [9, 14, 15], most probably explained due to an increased sympathetic tone. Furthermore, an increased number of out-of-hospital cardiac arrest [5, 6] during the COVID-19 pandemic and major cardiovascular complications including malignant VA in COVID-hospitalized patients have been described [1, 2]. Previous studies have also reported a seasonal accumulation of VA in ICD patients in times of high influenza-virus incidences [16].

As a consequence, one could assume an increased burden of VA during the COVID-19 pandemic in patients with an ICD, who are generally prone to the development of VA. In addition, most ICD patients are of elevated age, and therefore particularly at risk of severe COVID-19 disease [12].

However, the majority of authors investigating the influence of COVID-19 on the incidence of VA in ICD patients found a decrease or at least no increase of ICD therapies. O’Shea et al. [10] described a 32% reduction in adequate ICD therapies in 20 centers in the United States with an even more pronounced reduction (39%) in states with higher COVID-19 incidences. In a group of 455 ICD recipients in Bergamo, no change of VA burden was found during the COVID-19 outbreak (February 2020 to April 2020) in comparison to a control period in 2019 [11]. Sassone et al. [17] also found no increase of ICD therapies and a significant decrease of non-sustained VT during the first COVID lockdown in the province of Ferrara, Italy. Only Adabag et al. [13] described a significant rise of ICD therapies in New York City, New Orleans, and Boston between February and May 2020.

The authors assumed a more sedentary behavior due to extensive lockdown measures or home office regulations as the most probable cause of this observation that outbalances potential arrhythmogenic effects of COVID-19 as increased emotional stress, inflammation, or direct myocardial injury [10, 17]. This theory is supported by the findings of Galand et al. [12] who observed the distribution of ICD therapies during the first wave of COVID-19 in 30 French centers more precisely. The authors described a significant increase of ATP deliveries during the first weeks of the COVID spread, a period of presumable elevated emotional stress (first COVID case in France, increasing television coverage including the first French President television allocation). After the lockdown order and the 11 following weeks, there was significant drop of ICD therapies in France. According to the decrease of VA, the authors observed a significant reduction of mean heart rate which supports the theory of less sympathetic activity because of quarantine, social distancing, and/or home office leading to less ICD therapies.

In contrast to the abovementioned studies which focused on the first global COVID-19 outbreak (late 2019 and early 2020), we found a significant increase of appropriate ICD therapies during the second COVID-19 wave, a period of, at least in Germany, even higher COVID-19 incidences compared to early 2020 [18]. This observation may have several possible explanations. First, the higher degree of COVID-19 infections itself might cause a higher VA burden due to inflammation and direct myocardial damage. Even though the authors could not definitely determine this explanation, because the current infectious status of the patients developing VA was not available, we consider other factors more relevant. Hospitality and mortality rates were still lower in Germany in the observed period than during the first wave of COVID-19 in Italy or France where reduced VA rates have been described [19]. Therefore it seems unlikely that the higher infection rate alone can explain the rise of VA and ICD therapies.

COVID-19 vaccines were available in Germany from late 2020 and large governmental organized vaccination campaigns started in early 2021. As older people and patients with relevant comorbidities like heart failure were prioritized in the distribution, it can be assumed that a considerable proportion of the investigated collective has received a COVID-19 vaccine during the observation period. Similar to the infection itself, an association of cardiac complications including arrhythmias and COVID-19 vaccines has been described [20–22] which might also explain the increase of VA in the observed period. But comparable to the infectious status, we are not aware which patients were already vaccinated or if there was temporal association between vaccination and ICD therapies.

The general adherence to the lockdown measures and social distancing has declined throughout the pandemic [23]. As a consequence, the activity level of the observed ICD carriers might also have increased and could no longer outweigh the negative arrhythmogenic impact of the pandemic as chronic stress or direct inflammatory effects.

Most ICD patients suffer from an underlying cardiomyopathy requiring a strict regime of pharmacotherapy and fluid restriction which requires regular medical surveillance. The relationship between a rise of intrathoracic impedance reflecting fluid overload and risk of VA has been described in heart failure patients with an ICD [24]. There has been significant reduction of patient visits in all fields of cardiology services (inpatient and outpatient) especially in the first year of the pandemic [25]. The reasons for these reductions are multifactorial and include prioritization of medical services and especially patients’ reluctance to seek medical help due to fear of contracting the virus. A possible explanation for the difference of ICD therapies between the first and second COVID-19 wave might be a relative stable cardiac condition of patients with heart failure at the beginning of the pandemic that has worsened in the course of the year 2020 due to a reduction of medical surveillance.

## Limitations

This study has several potential limitations. First, it is limited by its retrospective character. Second, we are not aware of the infectious or vaccination status of the observed patients. Therefore, potential associations between infection, respectively, vaccination and an elevated risk of VA remain speculative. We did not systemically assess individual behavioral changes like physical activity, home office arrangement, or adherence to medical services; hence, the abovementioned correlations could not be proven as well. We found no significant difference of inappropriate ICD interventions during COVID-19 and the control period. However, the incidence of supraventricular tachycardia (e.g., atrial fibrillation) might differ between both groups, which would also be an interesting finding. As supraventricular arrhythmias are not routinely recorded in our ICD database (as long as they do not lead to inappropriate therapies), a systematic investigation of this question was not possible in this retrospective study. Similar considerations could be made regarding non-sustained VA. We solely observed the incidence of sustained VA requiring ICD interventions. Differences in the frequency of non-sustained VA might support or contradict our hypothesis about the relationship between COVID-19 and arrhythmia burden. Unfortunately, only VA requiring ICD interventions are routinely recorded in the database of our ambulatory clinic. Therefore, a systematic investigation of non-sustained VT was not possible in this retrospective study.

## Conclusion

In contrast to first COVID-19 spread in early 2020, which was characterized by a decrease or at least stable number of VA needing device therapies in most areas, we observed a significant rise of appropriate ICD therapies during the second wave of COVID-19 (November 2020–March 2021). Possible explanations for this observation include higher infectious rates, potential cardiac side effects of the vaccination as well as personal behavioral changes in physical activity or reduced utilization of medical services.
